# Variation in recommendations for management of pediatric fever and neutropenia across databases and languages: A systematic review of clinical practice guidelines

**DOI:** 10.1371/journal.pone.0361941

**Published:** 2026-11-03

**Authors:** Asher Hancock, Brandon Fickling, Tea Reljic, Gabrielle M. Haeusler, Vivian Moreno Berrio, Ambuj Kumar, Sheena Mukkada

**Affiliations:** 1 Department of Global Pediatric Medicine, St. Jude Children’s Research Hospital, Memphis, Tennessee, United States of America; 2 Research Methodology and Biostatistics Core, Morsani College of Medicine, University of South Florida, Tampa, Florida, United States of America; 3 Department of Infectious Diseases, Royal Children’s Hospital, Melbourne, Australia; 4 Murdoch Children’s Research Institute, Melbourne, Australia; 5 Department of Infectious Diseases, St. Jude Children’s Research Hospital, Memphis, United States of America; Stanford University, UNITED STATES OF AMERICA

## Abstract

**Background:**

Fever with neutropenia (FN) is a common complication of cancer therapy associated with significant morbidity, mortality, and resource use. Clinicians currently may access multiple Clinical Practice Guidelines (CPG) for FN in children to guide decision-making, however variation in content could impact care globally. We performed a systematic review and content analysis of CPG to assess the impact of databases searched and publication language on the quantity, quality, and content of recommendations identified for pediatric FN.

**Methods:**

We conducted a systematic search of PubMed, Embase, and Web of Science databases to identify evidence-based CPG published in the last 10 years addressing FN in children. We categorized CPG by language, extracted recommendations, characterized recommendation type (diagnostic, prognostic, or treatment), direction, and agreement with a recently developed international guideline. Methodological quality was assessed using the AGREE-II instrument and comparison between English and non-English CPG was performed using the Mann-Whitney U test. Comparisons of recommendation type (treatment, prognosis, diagnosis) and agreement with a reference guideline were assessed using the Chi-square test.

**Results:**

Fourteen CPG met inclusion criteria. Ten were published in English and four in non-English languages. There was no significant difference in methodological quality between language categories across AGREE-II domains except for scope and purpose (95.2% versus 90.5%; p = 0.036). Each database contained one unique guideline and only 36% (5/14) of the CPG were indexed in three databases. Percentage agreement between recommendations covered by 14 CPG and the reference CPG ranged between 33% and 100%.

**Conclusions:**

Using pediatric FN as an index topic, we found that language or database restrictions limit the quantity and content of recommendations. Since CPG have variable methodological quality regardless of source, clinicians should design appropriate search strategies based on context. We recommend a broad search strategy when healthcare context or disease prevalence differs across settings.

## Introduction

### Rationale

Fever with neutropenia (FN) is a common complication in cancer-directed therapy, occurring in up to 30% of neutropenic episodes in children with cancer [1,2]. FN can adversely impact quality of life, consume hospital resources, and be associated with significant morbidity if managed improperly [3]. Robustly developed clinical practice guidelines (CPG) play an important role in disseminating care based on the best available evidence and can inform clinician and patient decisions for a specific condition [4]. Nevertheless, the quality of CPG may differ, and this may be difficult for clinicians without guideline development knowledge to appreciate during the quick search necessary to provide care. It is also not well understood whether CPG developed for pediatric FN patients by different regional or professional groups address similar questions or make consistent recommendations. This may contribute to variation in practice and suboptimal patient outcomes.

Due to the increasing accessibility of peer-reviewed and non-peer-reviewed medical literature, developing guidance on how clinicians should navigate CPG selection is critical. When searching for CPG to inform clinical decisions, key questions include: 1) Can the search be restricted to a single database? 2) Will impactful information be lost if the search focuses only on CPG published in English? 3) Do topics addressed across CPG provide similar recommendations? The answers to these questions have not been well documented [5,6]. Using an index topic, management of FN in children, our study aimed to assess the impact of the quantity, quality, and content of CPG identified when restricting literature searches to a single database or English language publications.

## Materials and methods

We performed a systematic review of CPG following the Preferred Reporting Items for Systematic Reviews and Meta-Analysis (PRISMA), S1 Table[7]. A protocol for this study has not been registered. Ethics committee review was not applicable.

### Selection criteria

Any evidence-based CPG addressing the management of FN in pediatric patients in any language was eligible for inclusion (Fig 1). The search was restricted to publications within 10 years of the search date to prevent the inclusion of outdated recommendations.

**Fig 1:**
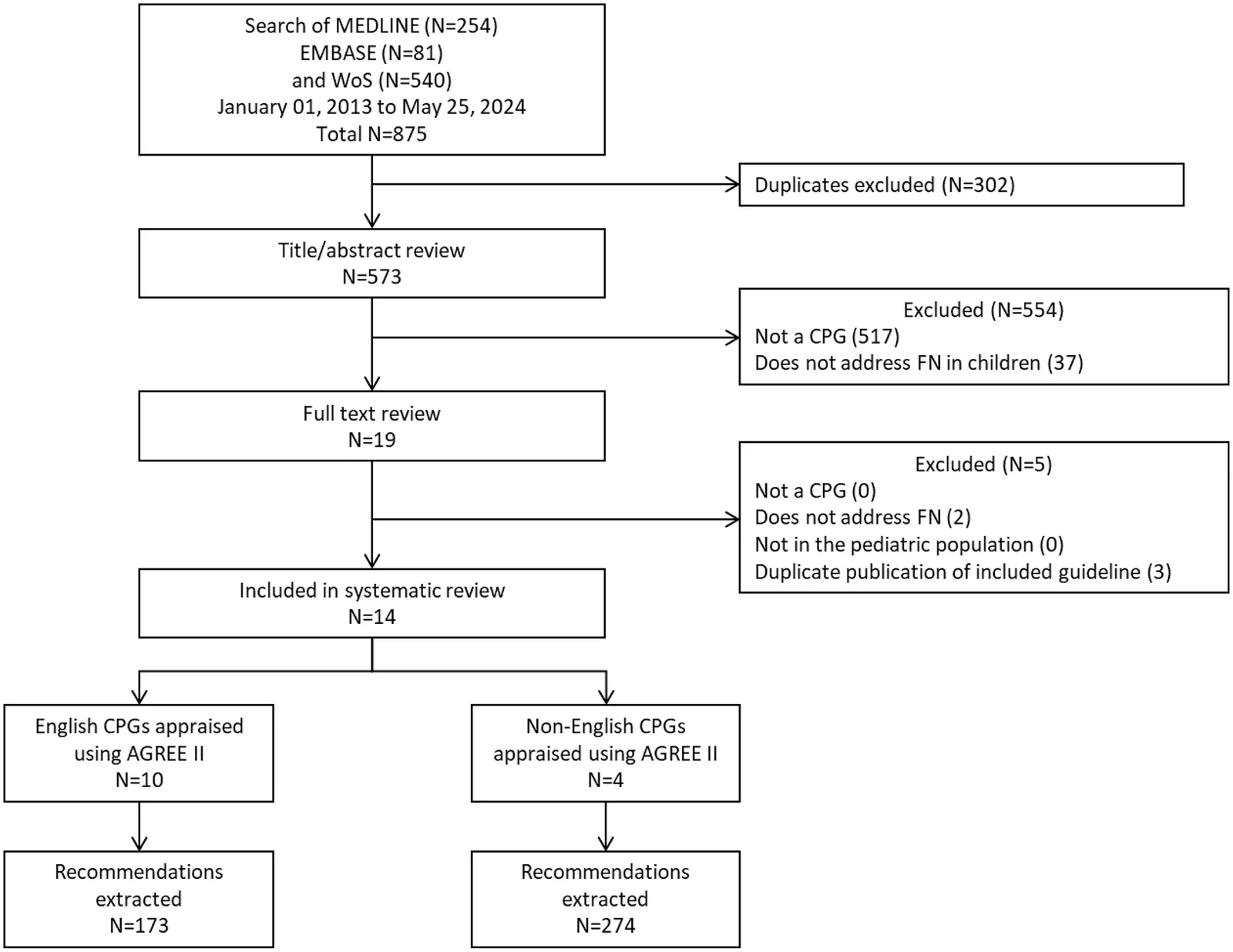
Flow chart (PRISMA diagram) depicting the search and selection of CPG.

### Search strategy

A systematic search of PubMed, Embase, and Web of Science databases between January 01, 2013, to May 25, 2024, using a combination of MeSH and free-text terms was performed to identify CPG. We searched for all clinical practice guidelines, regardless of language, in children, addressing the condition of febrile neutropenia. We used permutations of fever with neutropenia, febrile neutropenia, and applied“pediatric” “guideline” filters.The detailed search strategy for each database is provided in S2 Table.

### Selection process

Two reviewers independently screened all retrieved citations for inclusion in the study using the RAYYAN platform. To replicate a globally accessible strategy, Google Translate was used to translate non-English CPG into English. Any disagreements in the selections were resolved by consensus involving a third member of the study team.

### Primary and secondary outcomes

The primary outcome was the proportion of CPG and associated recommendations published in English versus non-English languages. The secondary outcomes were 1) the proportion of CPG and associated recommendations identified through PubMed versus additional databases, 2) comparison of methodological quality of English versus non-English-language CPG, 3) content analysis of recommendations across English and non-English guidelines stratified by guideline country of origin and country income level. The guideline country of origin was determined using the following hierarchy: 1) target geographic area specified in the guideline, 2) first author’s institutional affiliation country, 3) and/or corresponding author’s institutional affiliation. Country income level was categorized according to the 2024 World Bank classification system [8].

### Data collection

Recommendations from included CPG were extracted independently by two authors and reviewed by a third author. All inconsistencies were resolved through a consensus meeting. The unit of analysis for this systematic review was a guideline. Additionally, we extracted information on the country of origin, recommendation domain (treatment, prognosis, diagnosis), publication language, favorable versus unfavorable recommendation, and agreement with the recently developed resource-adapted ARIA FN Guide (www.ariaguide.org) [9]. These recommendations were used as a reference as they represent an amalgamation of recommendations rather than a single guideline reviewed in our evaluation.

### CPG appraisal

CPG were appraised using the Appraisal of Guidelines for Research & Evaluation Instrument II (AGREE-II) [10] with 23 appraisal items across 6 domains: scope and purpose, stakeholder involvement, rigor of development, clarity of presentation, applicability, and editorial independence. Two independent reviewers scored each question on a scale of 1–7, with 1 representing a high level of disagreement with the specified requirement. Appraisals were conducted independently by 2 reviewers using a Qualtrics form for each guideline mimicking the MYAGREE PLUS platform. A third author reviewed appraisals and disagreements were resolved in a joint meeting. Summary scores as percentages for each domain were obtained by summing the item scores in each domain and dividing by the highest possible score for the domain.

### Statistical analysis

The results were summarized using descriptive statistics. The differences between English versus non-English CPG were assessed using the Chi-square/Fisher exact test, as applicable, for categorical variables and Mann-Whitney U test for continuous variables. All analyses were performed using IBM SPSS statistical package version 30.

### Patient and public involvement

Patients and caregivers were not involved directly in this analysis of existing literature.

## Results

### Characteristics of included CPG

The initial search yielded 573 articles, of which 14 met the inclusion criteria (Figure 1). Ten CPG were in English and four in non-English languages (Spanish [n = 3] and German [n = 1]). Guideline development methodology was reported in 70% of English CPG and 100% of non-English CPG. The most frequently used methodology was Grading of Recommendations Assessment, Development and Evaluation (GRADE) (43%), while four CPG (29%) did not report any formal methodology. Seven (50%) guidelines originated from high-income countries (HIC), four (29%) from upper-middle income (UMIC), two (14%) from lower-middle income (LMIC), and one (7%) from low-income countries (LIC). Each database contained one unique CPG not included in the other databases while 36% (5/14) of the CPG were identified across all three databases. The characteristics of included CPG are summarized in Table 1.

**Table 1:** Characteristics of Included CPG. Data provided includes details of the authors, organizations, and publication details of included clinical practice guidelines.

Organization(s)	First Author	Corresponding Author	Language	Country	World Bank income classification	Scope	Year of Publication	Methodology	Database(s)	Update or Original Publication
AUNA Network	Alcarraz [11]	Gil-Olivares	Spanish	Peru	Upper-middle income	International	2021	GRADE	Web of Science	Original
Chilean Society of Infectology	Alvarez [12]	Alvarez	Spanish	Chile	Upper-middle income	International	2023	No methodology reported	EMBASE, Web of Science	Update
NICE	NICE [13]	NICE	English	United Kingdom	High income	International	2013	GRADE	PubMed, EMBASE	Original
SEIP and SEHOP	Campos [14]	Mendoza-Palomar	English	Spain	High income	International	2023	No methodology reported	Web of Science	Original
SWAB	de la Court [15]	de la Court	English	Netherlands	High income	National	2022	SWAB-AGREE II	PubMed, Web of Science	Original
SIOP and PODC	Israels [16]	Israels	English	Malawi	Low income	International	2013	No methodology reported	PubMed, EMBASE, Web of Science	Original
Indian Society of Critical Care Medicine	Kulkarni [17]	Kulkarni	English	India	Lower-middle income	National	2019	GRADE	PubMed, EMBASE	Original
ECIL-8	Lehrnbecher [18]	Groll	English	Germany	High income	International	2021	European Society of Clinical Microbiology and Infectious Diseases and European Confederation of Medical Mycology grading system	PubMed, EMBASE, Web of Science	Original
International Pediatric Fever and Neutropenia Guideline Panel	Lehrnbecher [19]	Sung	English	Canada	High income	International	2023	GRADE	PubMed, EMBASE, Web of Science	Update
Pediatric Hematology-Oncology (PHO) Chapter of Indian Academy of Pediatrics	Mehdi [20]	Bhat	English	India	Lower-middle income	International	2020	No methodology reported	EMBASE	Original
A multinational panel of Central American and Caribbean clinicians	Melgar [21]	Mukkada	English	United States	Upper-middle income	International	2020	GRADE	PubMed, EMBASE, Web of Science	Original
Committee on Infections in Immunocompromised Children of the Latin American Society of Pediatric Infectology	Santolaya [22]	Santolaya	Spanish	Chile	Upper-middle income	International	2021	GRADE	PubMed, Web of Science	Update
DGPI, GPOH, DGKJ, ÖGKJ, PIGS, and PEGeV	Simon [23]	Simon	German	Germany	High income	International	2024	AWMF Society Methodology	PubMed, EMBASE, Web of Science	Update
ASCO	Smith [24]	Smith	English	United States	High income	International	2015	GLIDES	PubMed	Update

Note: Grading of Recommendations Assessment, Development and Evaluation (GRADE); Guidelines Into Decision Support (GLIDES); National Institute for Clinical Excellence (NICE); Association of the Scientific Medical Societies in Germany (AWMF; Arbeitsgemeinschaft der Wissenschaftlichen Medizinischen Fachgesellschaften); Spanish Society of Pediatric Infectious Diseases (SEIP); Spanish Society of Pediatric Hematology and Oncology (SEHOP); Dutch Working Party on Antibiotic Policy (SWAB); International Society of Paediatric Oncology (SIOP); Paediatric Oncology in Developing Countries (PODC); 8th European Conference on Infections in Leukaemia (ECIL-8); German Society for Pediatric Infectious Diseases (DGPI); Society for Pediatric Oncology, Hematology (GPOH); German Society for Pediatric and Adolescent Medicine (DGKJ); Infectious Diseases Working Group of the Austrian Society for Pediatric and Adolescent Medicine (ÖGKJ); Pediatric Infectious Diseases Group Switzerland (PIGS), and the Paul Ehrlich Society for Chemotherapy (PEG eV); American Society of Clinical Oncology (ASCO)

### Methodological Quality Assessment

The median overall AGREE-II score was 85.7% (range: 42.9–100%) for English language CPG versus 71.4% (range: 71.4–85.7%) for non-English CPG (Table 2). The median score for guideline scope and purpose for English versus non-English language CPG was significantly different (95.2% versus 90.5%; p = 0.036). For all other domains, there was no significant difference between English versus non-English language CPG. The AGREE-II appraisals by domain for each guideline are available in S3 Table.

**Table 2:** Summary of AGREE-II Appraisal Scores.

Domain	Overall Score	English CPG	Non-English CPG
Scope and purpose	95.2% (81.0-100%)	95.2% (90.5-100%)	90.5% (81.0-95%)
Stakeholder involvement	69.0% (52.4-100%)	73.8% (52.4-100%)	57.1% (52.4-81.0%)
Rigor of development	79.5% (44.6-96.4%)	84.8% (44.6-96.4%)	74.1% (69.6-91.1%)
Clarity of presentation	90.5% (57.1-100%)	90.5% (57.1-100%)	88.1% (71.4-100%)
Applicability	66.1% (57.1-100%)	69.6% (57.1-100%)	58.9% (57.1-67.9%)
Editorial independence	92.9% (35.7-100%)	92.9% (35.7-100%)	71.4% (42.9-100%)
Overall score	78.6% (42.9-100%)	85.7% (42.9-100%)	71.4% (71.4-85.7%)

Note: The percentages and ranges here represent the percent scores for each domain, where a score of 100% indicates the highest quality for that domain or overall quality. A score below 100% indicates deficiencies in the conduct or reporting of a CPG.

### Characteristics of recommendations

The median number of recommendations was 24 (range: 2–107); 19.5 (range: 2–30) for English CPG and 69.5 (range: 28–107) for non-English CPG. There was a significant difference (p = 0.003) in the number of recommendations by domain (diagnostic, prognostic, or treatment) between languages (Table 3).

**Table 3:** Analysis of recommendation type, direction, and alignment across CPG.

Recommendation Category	Recommendation Sub-Category	English n (%)	Non-English n (%)	p-value
Type	Diagnostic	40 (23)	91 (33)	p = 0.003
	Prognosis	11 (6)	4 (2)	
	Treatment	122 (71)	179 (65)	
	Total	173	274	
Direction	Against	31 (18)	24 (9)	p = 0.005
	For	142 (82)	250 (91)	
	Total	173	274	
Alignment with ARIA	No	8 (8)	18 (16)	p = 0.077
	Yes	93 (92)	96 (84)	
	Total	101	114	

Note: Recommendations not included in ARIA were excluded from the analysis for alignment.

### Distribution of favorable versus unfavorable recommendations

As shown in Table 3, there was a significant difference in the percentage of recommendations worded as favorable (for/recommend) versus unfavorable (against/do not recommend) between English and non-English language CPG (82% favorable in English versus 91% in non-English; p = 0.005).

### Content analysis of recommendations

#### Definition of fever.

Fever definitions were reported in four of the fourteen guidelines (29%) [13–15]. Temperature thresholds for a single measurement of fever ranged from 38◦C to 38.5◦C [13–15]. Two CPG specified lower thresholds when taking multiple axillary measurements within an hour [14,15].

#### Initial diagnostics: Urinalysis.

Six of the fourteen CPG (43%) recommended urinalysis in the initial diagnostic workup for pediatric FN [11,12,15,19,21,22]. Of these, two originated from HIC (33%) and four UMIC (67%). Routine urinalysis was recommended for pediatric FN in two (UMIC) CPG [11,21], while three (1 HIC, 2 UMIC) of the six recommended urinalysis only when a clean catch is readily available [12,19,22], and one (HIC) recommended urinalysis when a urinary tract infection is suspected or there is a history of recurrent urinary tract infections [15].

#### Initial diagnostics: Blood cultures.

Eleven out of fourteen (79%) CPG had recommendations on initial diagnostic assessment using blood cultures. Five were from HIC (45%), four UMIC (36%), one LMIC (9%), and one LIC (9%) [11]. Of these, six (2 HIC, 3 UMIC, 1 LMIC) recommended obtaining concurrent central and peripheral sets of blood cultures [12,15,17,19,21,22], while one (HIC) recommended avoiding a peripheral culture and exclusively obtaining cultures from the central line [23].

#### Initial diagnostics: Chest X-ray.

Eight CPG (57%) addressed the use of chest X-ray and recommended against its use unless there are respiratory signs or symptoms that could suggest a pulmonary focus [11–13,15,19,21–23]. Of these, half were from HIC and half from UMIC.

### Tropical pathogens

Two of fourteen CPG (14%) made recommendations addressing the diagnosis or management of tropical pathogens [16,17]. Of these, one (LIC) CPG recommended thick blood films to be used in areas where malaria is endemic [16], and one (LMIC) CPG recommended against empirically treating all patients presenting with FN for tropical infections, specifically naming malaria and leptospirosis [17].

### Risk stratification

Seven of the fourteen CPG (50%) recommended the use of a risk stratification tool to manage pediatric FN [12–14,18,19,21,22]. Of these, four were from HIC (57%) and three from UMIC (43%). Only one of the seven CPG, from an UMIC, recommended a specific risk stratification schema: the PINDA stratification model [12]. One (HIC) recommended against the use of a risk stratification tool in managing pediatric FN due to insufficient evidence [15].

### Initial empiric antibiotic therapy recommendations

Six of the fourteen CPG (43%) employed a risk-stratified approach to guide initial empiric treatment; half were from HIC and half from UMIC [12,14,19,21–23]. For high-risk FN, three (1 UMIC, 2 HIC) recommended monotherapy with an antipseudomonal beta-lactam [14,19,21]. One (HIC) recommended combination therapy with an antipseudomonal beta-lactam and a second agent based on individual risk factors [23], and two (UMIC) recommended either monotherapy with an antipseudomonal beta-lactam or combination therapy with ceftazidime and an aminoglycoside [12,22]. All six CPG recommended an additional broad-spectrum antibiotic in patients who are high-risk and hemodynamically unstable [12,14,19,21–23].

Seven CPG addressed management of low-risk FN [12,14,18,19,21–23]. Three (1 HIC, 2 UMIC) CPG recommended inpatient monitoring for up to 48 hours [12,14,22], two (1 HIC, 1 UMIC) CPG recommended outpatient monitoring if the infrastructure was suitable for close follow-up [19,21], and one (HIC) CPG did not specify a setting [23]. Three CPG specified antibiotic regimens for low-risk FN: one (UMIC) recommended a beta-lactam without antipseudomonal activity or ciprofloxacin [12], one (HIC) recommended only a third-generation cephalosporin without antipseudomonal activity [22], and one (HIC) recommended an antipseudomonal beta-lactam [23].

### Invasive fungal disease: empiric therapy and agents

Recommendations for when to initiate antifungal therapy and/or the first line agents were included in nine of the fourteen CPG (64%) [11,12,14,15,17,19,21–23]. Of these, four CPG were from HIC (44%), four from UMIC (44%), and one from LMIC (11%). Six CPG contained a recommendation addressing preemptive therapy. One (HIC) CPG recommended preemptive therapy in non-HCT, high-risk patients, not receiving anti-mold prophylaxis and with prolonged neutropenia unresponsive to antibiotics [19], three (1 HIC, 2 UMIC) recommended preemptive therapy over empiric therapy if resources permit [12,15,22], and two (HIC) endorsed preemptive and empiric therapy as equally suitable options [14,23]. Three (2 UMIC, 1 LMIC) CPG recommended empiric antifungal therapy without addressing preemptive strategies [11,17,21]. Nine CPG addressed preemptive or empiric therapy and recommended first line agents for patients suspected of or at high risk of invasive fungal disease. CPG from UMIC and HIC recommended caspofungin or liposomal amphotericin B as first line, whereas the single LMIC CPG recommended an echinocandin without specifying an agent [17].

### Invasive Fungal Disease: Diagnostics

Six of the fourteen CPG (43%) contained recommendations regarding the use of galactomannan or beta-D-glucan for the diagnosis of invasive fungal disease in pediatric FN. Two were from HIC (33%), three from UMIC (50%), and one from LMIC (17%). Galactomannan was recommended in four (1 HIC, 2 UMIC, 1 LMIC) CPG while two (1 HIC, 1 UMIC) recommended against its routine use [12,17,19,21–23]. One (LMIC) CPG recommended using beta-D-glucan while three (1 HIC, 2 UMIC) CPG recommended against its routine use [12,17,19,22].

### Agreement with ARIA recommendations across included CPG

Of the English-language recommendations, 58% (101/173) were addressed in ARIA versus 42% (114/274) of non-English recommendations. Overall, of the 215 recommendations addressed in ARIA, 189 (88%) aligned with the ARIA Guide’s FN recommendations. There was no significant difference in agreement with ARIA recommendations between CPG published in English and non-English language (p = 0.077; Table 3). Agreement with ARIA recommendations, depending on the covered topic, ranged from a high of 100% to a low of 33% (Table 4).

**Table 4:** Agreement with ARIA recommendations across included CPG.

Reference topics	Alcarraz (2021)	Alvarez (2023)	NICE (2013)	Campos (2023)	de la Court (2022)	Israels (2013)	Kulkarni (2019)	Lehrnbecher (2021)	Lehrnbecher (2023)	Mehdi (2020)	Melgar (2021)	Santolaya (2021)	Simon (2024)	Smith (2015)	Topics Covered (%)	ARIA agreement (%)	Agreement between guidelines (%)
Urinalysis	YES	YES	–	–	NO	–	–	–	YES	–	YES	YES	–	–	43	83	83
Blood Cultures	YES	YES	YES	YES	YES	YES	YES	–	YES	–	YES	YES	YES	–	79	100	100
Chest X-ray	YES	YES	YES	–	YES	–	–	–	YES	–	YES	YES	YES	–	57	100	100
Risk Stratification	–	YES	YES	YES	NO	–	–	YES	YES	–	YES	YES	–	–	57	88	88
Initial Management	YES	YES	YES	YES	YES	NO	YES	YES	YES	–	YES	YES	YES	–	86	92	92
Prolonged Fever	NO	–	NO	YES	YES	NO	–	YES	YES	–	YES	–	YES	–	64	67	67
Deescalation	YES	YES	–	YES	YES	–	NO	YES	YES	–	YES	YES	YES	–	71	90	90
Escalation	YES	YES	–	YES	YES	YES	YES	YES	YES	–	YES	YES	–	–	71	100	100
Fungal Diagnostics	–	NO	–	NO	–	–	NO	–	YES	–	YES	YES	NO	–	50	43	43
Empiric Antifungal Therapy	YES	YES	–	YES	NO	–	YES	–	YES	–	YES	YES	YES	–	64	89	89
Cessation of Treatment	–	YES	YES	YES	YES	–	–	YES	YES	–	YES	YES	YES	–	64	100	100
Discharge	–	NO	YES	YES	–	–	–	–	–	–	YES	NO	NO	–	43	50	50
G-CSF	–	–	–	NO	YES	–	–	–	–	–	–	–	–	NO	21	33	33

Dash illustrates the guideline does not make a recommendation on the topic.

YES indicates agreement with ARIA recommendations.

NO indicates disagreement with ARIA recommendations.

## Discussion

Our systematic review yielded several findings with important implications for clinical practice. CPG for pediatric FN are indexed across multiple databases, each yielding at least one unique guideline. Most CPG were published in English and created in HIC or UMIC. Nevertheless, the methodological quality of CPG in English versus non-English languages did not differ significantly. Regardless of language, most CPG focused on interventions rather than diagnosis or prognosis. A significant proportion (86.5% on average) of the recommendations focused on the favorability of an intervention, with limited recommendations against management practices that are unfavorable and/or not indicated. Overall, recommendations agreed with the recently developed ARIA Guide with no significant difference between English and non-English CPG. However, the results from the content analysis of the CPG stratified by income settings—according to the World Bank Classification—showed variability across clinical recommendations in terms of diagnostic approaches and treatment strategies. All CPG had at least one unique recommendation not covered in other CPG.

As pediatric FN must be treated across global settings, these findings have implications for clinical practice. Our findings suggest that—regardless of database or language—CPG tended to contain similar methodologic rigor and quality with mixed overall agreement in clinical topics. The heterogeneity of recommendations and the wide range of agreement across CPG with the ARIA Guide signals the complexity of identifying consistent recommendations for conditions, such as pediatric FN, that may have significant heterogeneity in how they are managed across resource settings. Additionally, context-specific recommendations that are guided by local epidemiology and resources may be lost with a restricted search.

In the content analysis, specifically addressing CPG diagnostics, the most recommended diagnostic intervention, regardless of income setting, was blood cultures; however, not all recommendations contained specific guidelines concerning central versus peripheral cultures. This reflects panel priorities and may be impacted by varying prevalence of central lines, cost considerations, and provider or patient/family preferences. Certain diagnostic interventions, specifically urinalysis and chest X-ray recommendations, were only present in UMIC/HIC CPG. Others, such as recommendations regarding galactomannan and beta-D-glucan to diagnose invasive fungal disease, did not correlate with income level; each test was endorsed and explicitly opposed in separate HIC CPG, while the single LMIC CPG recommended both tests, which could be attributed to available resources [17]. While the management of tropical pathogens was discussed only in LIC/LMIC CPG, this difference is likely driven by differences in endemicity of disease, rather than resource level [16,17]. Overall, these findings highlight that medical decision-making is complex, and that recommendations are shaped by epidemiology and resource availability, which ideally translates into resource-specific clinical care, tailored to local circumstances [25].

Additionally, when comparing treatment strategies, we discovered that the guidelines from HIC and UMIC tended to recommend a pre-emptive approach for antifungal therapy in high-risk patients, whereas the single guideline from LMIC/LIC recommended empiric antifungal treatment [17]. We analyzed CPG content across a decade, a period during which we did not expect evidence to change for many topics; however, research and practice updates may have resulted in temporal shifts across CPG for selected topics. This could have impacted recommendations particularly for antifungal therapy, where all recommendations using a preemptive therapy approach originated after 2021. Nevertheless, since a preemptive approach requires access to diagnostic studies and imaging, limitations in resources by setting rather than evidence against their use may be the decisive factor in clinical management. Concerning antibiotics, only HIC/UMIC CPG addressed first-line antibiotic regimens or appropriate settings to manage low-risk FN, likely reflecting fewer options for antibiotic regimens and differences in the capacity to treat low-risk patients. There was overall consistency across recommendations in using an antipseudomonal antibiotic for high-risk FN. However, the appropriate setting to monitor low-risk FN—whether inpatient or outpatient—and suitable antibiotics varied [15,18,22]. These differences in recommendations across income settings underscore the utility of a broad search approach to inform recommendations tailored to resources and epidemiology. The interpretation of identical evidence by clinicians is shaped by local factors—including cost, quality, and access—that may ultimately alter the resulting clinical decision-making [26].

### Limitations

While this systematic review illustrates important findings, it has a few limitations. We initiated the project as a quality improvement activity and did not register the study protocol in a public domain. The search for pediatric FN CPG from three databases could have underestimated the number of non-English CPG as there are databases in languages other than English. However, our goal was to assess the prevalence of CPG indexed in the most commonly used databases using a consistent strategy, making this approach appropriate for the question we posed [27]. Our analysis considered CPG across a 10-year period during which evidence and availability of diagnostic and therapeutic interventions may have changed. Nevertheless, recommendations were more aligned than conflicting across CPG, so this effect appeared limited to certain recommendations. This effect may also have been mitigated by panel values and preferences, which are governed by factors beyond evidence. Newer guidelines may have either better or worse alignment; this remains to be addressed in future studies. Another minor limitation of our study is the lack of ability to be prescriptive when it comes to assessing the methodological quality of English versus non-English CPG. AGREE II does not define absolute cut-offs for “high” or “low” quality CPG. While a threshold for quality is desirable, it may not be feasible because high scores on one domain cannot be a substitute for a lower scores on other domains. Therefore, the current AGREE II approach provides transparency for decision-making and leaves it to the end user to determine usability of the CPG. Finally, our study focused only on pediatric FN; therefore, the findings may or may not apply to other topics. Nevertheless, our study provides initial evidence of the significance of setting and disease context when searching for CPG.

## Conclusion

In summary, this study of pediatric FN CPG shows that limiting a search for CPG by language or database may limit both the quantity and content of recommendations identified. Since methodological rigor has no correlation with language or databases searched, it is important to train clinicians to critically evaluate how recommendations are generated. The variability in recommendations between geographic and income settings is understandable but highlights the need for resource-adapted CPG that recognize differences in values and preferences of users to provide recommendations that are suitable across clinical settings. As quality is not uniform, clinicians must be trained to search for CPG and critically appraise them to make the most appropriate decisions based on methodologically sound CPG.

## Supporting information

S1 Table PRISMA Checklist. (DOCX)

S2 Table Full Search Strategies Employed to Identify CPG. (DOCX)

S3 Table AGREE II appraisals by domain for guidelines on the management of pediatric fever and neutropenia using the AGREE II checklist. (DOCX)

## Abbreviations

CPG: Clinical practice guidelines
FN: Fever with neutropenia
ARIA: Adapted Resource and Implementation Application
SIOP: International Society of Paediatric Oncology
PRISMA: Preferred Reporting Items for Systematic Reviews and Meta-Analysis
AGREE-II: Appraisal of Guidelines for Research & Evaluation Instrument II
GLIDES: Guidelines Into Decision Support
HIC: high-income country
UMIC: upper-middle income countries
LMIC: lower-middle income countries
LIC: low-income countries
