# Supplementary material for: Variation in recommendations for management of pediatric fever and neutropenia across databases and languages: A systematic review of clinical practice guidelines
Source: PLoS One. 2026 Nov 3;21(11):e0361941. doi: 10.1371/journal.pone.0361941 (PMC13730364; doi:10.1371/journal.pone.0361941)
Supplement: Supplementary file 2 [file pone.0361941.s002.docx]

S1 Table: Full Search Strategies Employed to Identify CPG

**PubMed search strategy**

| No. | Query | Item |
| --- | --- | --- |
| 1 | "Febrile Neutropenia"[MeSH Terms] OR "Febrile Neutropenia"[Title/Abstract] OR "Fever and Neutropenia"[Title/Abstract] | Febrile neutropenia search |
| 2 | "infant"[MeSH Terms] OR "infant*"[All Fields] OR ("infancy"[Journal] OR "infancy"[All Fields]) OR "newborn*"[All Fields] OR "baby*"[All Fields] OR ("baby s"[All Fields] OR "babys"[All Fields] OR "infant"[MeSH Terms] OR "infant"[All Fields] OR "babies"[All Fields]) OR "neonat*"[All Fields] OR "preterm*"[All Fields] OR "prematur*"[All Fields] OR "postmatur*"[All Fields] OR "child"[MeSH Terms] OR "child*"[All Fields] OR "schoolchild*"[All Fields] OR (("educational status"[MeSH Terms] OR ("educational"[All Fields] AND "status"[All Fields]) OR "educational status"[All Fields] OR "schooling"[All Fields] OR "education"[MeSH Terms] OR "education"[All Fields] OR "school s"[All Fields] OR "schooled"[All Fields] OR "schools"[MeSH Terms] OR "schools"[All Fields] OR "school"[All Fields]) AND "age"[All Fields]) OR "preschool*"[All Fields] OR "Kid"[All Fields] OR "kids"[All Fields] OR "toddler*"[All Fields] OR "adolescent"[MeSH Terms] OR "adoles*"[All Fields] OR "teen*"[All Fields] OR "boy"[All Fields] OR "girl*"[All Fields] OR "minors"[MeSH Terms] OR "minors*"[All Fields] OR "puberty"[MeSH Terms] OR "pubert*"[All Fields] OR "pubescen*"[All Fields] OR "prepubescen*"[All Fields] OR "pediatrics"[MeSH Terms] OR "pediatric*"[All Fields] OR "paediatric*"[All Fields] OR "peadiatric*"[All Fields] OR "schools"[MeSH Terms] OR (("nurseries, infant"[MeSH Terms] OR ("nurseries"[All Fields] AND "infant"[All Fields]) OR "infant nurseries"[All Fields] OR "nurseries"[All Fields] OR "nursery"[All Fields]) AND "school*"[All Fields]) OR "kindergar*"[All Fields] OR (("primaries"[All Fields] OR "primary"[All Fields]) AND "school*"[All Fields]) OR (("neoplasm metastasis"[MeSH Terms] OR ("neoplasm"[All Fields] AND "metastasis"[All Fields]) OR "neoplasm metastasis"[All Fields] OR "secondaries"[All Fields] OR "secondary"[MeSH Subheading] OR "secondary"[All Fields]) AND "school*"[All Fields]) OR ("Elementary"[All Fields] AND "school*"[All Fields]) OR ("High"[All Fields] AND "school*"[All Fields]) OR "highschool*"[All Fields] | Pediatric filter |
| 3 | ("critical pathways"[MeSH Terms:noexp] OR "clinical protocols"[MeSH Terms:noexp] OR "consensus"[MeSH Terms:noexp] OR "consensus development conference"[Publication Type] OR "Consensus Development Conferences as Topic"[MeSH Terms] OR "guideline"[Publication Type] OR "practice guideline"[Publication Type] OR "guidelines as topic"[MeSH Terms:noexp] OR "practice guidelines as topic"[MeSH Terms:noexp] OR "health planning guidelines"[MeSH Terms:noexp] OR "consensus development conference, nih"[Publication Type:noexp] OR ("position statement*"[Title/Abstract] OR "position statement*"[Other Term] OR "position statement*"[Other Term] OR "guidance statement*"[Title/Abstract] OR "policy statement*"[Title/Abstract] OR "policy statement*"[Other Term] OR "policy statement*"[Other Term] OR "practice parameter*"[Title/Abstract] OR "practice parameter*"[Other Term] OR "practice parameter*"[Other Term] OR "best practice*"[Title/Abstract] OR "best practice*"[Other Term] OR "best practice*"[Other Term]) OR ("standards"[Title] OR "standards"[Other Term] OR "guideline"[Title] OR "guideline"[Other Term] OR "guidelines"[Title] OR "guidelines"[Other Term]) OR ("practice guideline*"[Title/Abstract] OR "treatment guideline*"[Title/Abstract] OR "clinical guideline*"[Title/Abstract]) OR ("CPG"[Title] OR "CPGs"[Title]) OR ("consensus*"[Title/Abstract] OR "consensus*"[Other Term]) OR (("critical"[Title/Abstract] OR "critical"[Other Term] OR ("clinical"[Title/Abstract] OR "clinical"[Other Term])) AND ("practice"[Title/Abstract] OR "practice"[Other Term]) AND ("path"[Title/Abstract] OR "path"[Other Term] OR ("paths"[Title/Abstract] OR "paths"[Other Term]) OR ("pathway"[Title/Abstract] OR "pathway"[Other Term]) OR ("pathways"[Title/Abstract] OR "pathways"[Other Term]) OR ("protocol*"[Title/Abstract] OR "protocol*"[Other Term]))) OR ("recommendat*"[Title] OR "recommendat*"[Other Term]) OR (("care"[Title/Abstract] OR "care"[Other Term]) AND ("standard"[Title/Abstract] OR "standard"[Other Term] OR ("path"[Title/Abstract] OR "path"[Other Term]) OR ("paths"[Title/Abstract] OR "paths"[Other Term]) OR ("pathway"[Title/Abstract] OR "pathway"[Other Term]) OR ("pathways"[Title/Abstract] OR "pathways"[Other Term]) OR ("map"[Title/Abstract] OR "map"[Other Term]) OR ("maps"[Title/Abstract] OR "maps"[Other Term]) OR ("plan"[Title/Abstract] OR "plan"[Other Term]) OR ("plans"[Title/Abstract] OR "plans"[Other Term]))) OR (("algorithm*"[Title/Abstract] OR "algorithm*"[Other Term]) AND ("screening"[Title/Abstract] OR "screening"[Other Term] OR ("examination"[Title/Abstract] OR "examination"[Other Term]) OR ("test"[Title/Abstract] OR "test"[Other Term]) OR ("tested"[Title/Abstract] OR "tested"[Other Term]) OR ("testing"[Title/Abstract] OR "testing"[Other Term]) OR ("assessment*"[Title/Abstract] OR "assessment*"[Other Term]) OR ("diagnosis"[Title/Abstract] OR "diagnosis"[Other Term]) OR ("diagnoses"[Title/Abstract] OR "diagnoses"[Other Term]) OR ("diagnosed"[Title/Abstract] OR "diagnosed"[Other Term]) OR ("diagnosing"[Title/Abstract] OR "diagnosing"[Other Term]))) OR (("algorithm*"[Title/Abstract] OR "algorithm*"[Other Term]) AND ("pharmacotherap*"[Title/Abstract] OR "pharmacotherap*"[Other Term] OR ("chemotherap*"[Title/Abstract] OR "chemotherap*"[Other Term]) OR "chemotreatment*"[Title/Abstract] OR ("therap*"[Title/Abstract] OR "therap*"[Other Term]) OR ("treatment*"[Title/Abstract] OR "treatment*"[Other Term]) OR ("intervention*"[Title/Abstract] OR "intervention*"[Other Term])))) | Guideline filter |
| 4 | #1 AND #2 AND #3 |  |

**EMBASE search strategy**

| No. | Query | Item |
| --- | --- | --- |
| 1 | 'febrile neutropenia'/exp OR 'febrile neutropenia' OR 'fever and neutropenia' | Febrile neutropenia search |
| 2 | 'child'/exp OR 'congenital, hereditary, and neonatal diseases and abnormalities'/exp OR 'infant'/exp OR 'adolescence'/exp OR 'infant, newborn'/exp OR 'child, preschool'/exp OR ((((((pediatric*:kw OR paediatric*:kw OR child*:kw OR newborn*:kw OR congenital*:kw OR infan*:kw OR baby:kw OR babies:kw OR neonat*:kw OR 'pre term':kw OR premature:kw) AND birth:kw OR nicu:kw OR preschool*:kw OR kindergarten*:kw OR elementary:kw) AND school*:kw OR nursery:kw) AND school*:kw OR schoolchild*:kw OR toddler*:kw OR boy:kw OR boys:kw OR girl*:kw OR middle:kw) AND school*:kw OR pubescen*:kw OR juvenile*:kw OR teen*:kw OR youth*:kw OR high:kw) AND school*:kw) OR adolesc*:kw OR 'pre pubesc*':kw OR child*:ta OR adolesc*:ta OR pediat*:ta OR paediat*:ta | Pediatric filter |
| 3 | 'guideline':it OR 'practice guideline':it OR 'consensus development conference':it OR 'consensus development conference, nih':it OR 'guideline*':ti OR 'standards':ti OR 'consensus*':ti OR 'recommendat*':ti OR 'practice parameter*':ti OR 'position statement*':ti OR 'guidance statement*':ti OR 'practice bulletin*':ti OR 'policy statement*':ti OR 'cpg':ti OR 'cpgs':ti OR 'best practice*':ti OR ('care':ti AND ('path':ti OR 'paths':ti OR 'pathway':ti OR 'pathways':ti OR 'map':ti OR 'maps':ti OR 'plan':ti OR 'plans':ti OR 'standard':ti)) OR (('critical':ti OR 'clinical':ti OR 'practice':ti) AND ('path':ti OR 'paths':ti OR 'pathway':ti OR 'pathways':ti OR 'protocol*':ti)) OR ('algorithm*':ti AND ('pharmacotherap*':ti OR 'chemotherap*':ti OR 'chemotreatment*':ti OR 'therap*':ti OR 'treatment*':ti OR 'intervention*':ti)) OR ('algorithm*':ti AND ('screening':ti OR 'examination':ti OR 'test':ti OR 'tested':ti OR 'testing':ti OR 'assessment*':ti OR 'diagnosis':ti OR 'diagnoses':ti OR 'diagnosed':ti OR 'diagnosing':ti)) OR 'guideline*':kw OR 'standards':kw OR 'consensus*':kw OR 'recommendat*':kw OR 'practice parameter*':kw OR 'position statement*':kw OR 'practice bulletin*':kw OR 'policy statement*':kw OR 'cpg':kw OR 'cpgs':kw OR 'best practice*':kw OR ('care':kw AND ('path':kw OR 'paths':kw OR 'pathway':kw OR 'pathways':kw OR 'map':kw OR 'maps':kw OR 'plan':kw OR 'plans':kw OR 'standard':kw)) OR (('critical':kw OR 'clinical':kw OR 'practice':kw) AND ('path':kw OR 'paths':kw OR 'pathway':kw OR 'pathways':kw OR 'protocol*':kw)) OR ('algorithm*':kw AND ('pharmacotherap*':kw OR 'chemotherap*':kw OR 'therap*':kw OR 'treatment*':kw OR 'intervention*':kw)) OR ('algorithm*':kw AND ('screening':kw OR 'examination':kw OR 'test':kw OR 'tested':kw OR 'testing':kw OR 'assessment*':kw OR 'diagnosis':kw OR 'diagnoses':kw OR 'diagnosed':kw OR 'diagnosing':kw)) OR (('systematic review':ti OR 'systematic review':it OR 'systematic review':kw) AND ('practice guideline*':ti,ab OR 'treatment guideline*':ti,ab OR 'clinical guideline*':ti,ab OR 'guideline recommendation*':ti,ab)) | Guideline filter |
| 4 | #1 AND #2 AND #3 |  |

**Web of Science search strategy**

| No. | Query | Item |
| --- | --- | --- |
| 1 | ALL=("Febrile Neutropenia" OR "Fever and Neutropenia") | Febrile neutropenia search |
| 2 | (((((AK=(child)) OR AK=(infant)) OR AK=(adolescent)) OR AK=(newborn)) OR AK=(pediatric)) OR AK=(neonatal) OR TS=(pediatric* OR paediatric* OR child* OR newborn* OR congenital* OR infan* OR baby OR babies OR neonat* OR 'pre term' OR premature) OR TS=(adolesc* OR 'pre$pubesc*' OR child* OR adolesc* OR pediat* OR paediat*) OR TS=(nursery OR schoolchild* OR toddler* OR boy OR boys OR girl* OR pubescen* OR juvenile* OR teen* OR youth*) | Pediatric filter |
| 3 | TI=('guideline' OR 'practice guideline' OR 'consensus development conference' OR 'consensus development conference, nih' OR 'guideline*' OR 'standards' OR 'consensus*' OR 'recommendat*' OR 'practice parameter*' OR 'position statement*' OR 'guidance statement*' OR 'practice bulletin*' OR 'policy statement*' OR 'cpg' OR 'cpgs' OR 'best practice*') OR (TI=('care')) AND TI=('path' OR 'paths' OR 'pathway' OR 'pathways' OR 'map' OR 'maps' OR 'plan' OR 'plans' OR 'standard'') OR (TI=('critical' OR 'clinical' OR 'practice')) AND TI=('path' OR 'paths' OR 'pathway' OR 'pathways' OR 'protocol*') OR (TI=('algorithm*')) AND TI=('pharmacotherap*' OR 'chemotherap*' OR 'chemotreatment*' OR 'therap*' OR 'treatment*' OR 'intervention*' OR 'screening' OR 'examination' OR 'test' OR 'tested' OR 'testing' OR 'assessment*' OR 'diagnosis' OR 'diagnoses' OR 'diagnosed' OR 'diagnosing'') OR TS=('guideline*' OR 'standards' OR 'consensus*' OR 'recommendat*' OR 'practice parameter*' OR 'position statement*' OR 'practice bulletin*' OR 'policy statement*' OR 'cpg' OR 'cpgs' OR 'best practice*') OR (TI=('systematic review')) AND TS=('practice guideline*' OR 'treatment guideline*' OR 'clinical guideline*' OR 'guideline recommendation*'') | Guideline filter |
| 4 | #1 AND #2 AND #3 |  |
