# Supplementary material for: Variation in recommendations for management of pediatric fever and neutropenia across databases and languages: A systematic review of clinical practice guidelines
Source: PLoS One. 2026 Nov 3;21(11):e0361941. doi: 10.1371/journal.pone.0361941 (PMC13730364; doi:10.1371/journal.pone.0361941)
Supplement: Supplementary file 3 [file pone.0361941.s003.docx]

S2 Table: AGREE II appraisals by domain for guidelines on the management of pediatric fever and neutropenia using the AGREE II checklist

| **Guideline** | **Scope and purpose** | **Stakeholder involvement** | **Rigor of development** | **Clarity of presentation** | **Applicability** | **Editorial Independence** | **Overall** |
| --- | --- | --- | --- | --- | --- | --- | --- |
| Alcarraz 2021 | 81% | 62% | 91% | 90% | 57% | 100% | 71% |
| Alvarez 2023 | 90% | 52% | 70% | 100% | 57% | 43% | 71% |
| NICE 2013 | 100% | 100% | 96% | 100% | 100% | 93% | 100% |
| Campos 2023 | 95% | 52% | 45% | 86% | 68% | 100% | 57% |
| de la Court 2022 | 100% | 76% | 93% | 95% | 57% | 86% | 86% |
| Israels 2013 | 90% | 71% | 57% | 57% | 75% | 93% | 57% |
| Kulkarni 2019 | 95% | 67% | 59% | 90% | 64% | 86% | 71% |
| Lehrnbecher 2021 | 95% | 62% | 91% | 100% | 71% | 93% | 86% |
| Lehrnbecher 2023 | 95% | 90% | 96% | 90% | 64% | 100% | 100% |
| Mehdi 2020 | 95% | 57% | 50% | 71% | 57% | 36% | 43% |
| Melgar 2020 | 95% | 76% | 84% | 100% | 75% | 93% | 100% |
| Santolaya 2021 | 95% | 52% | 73% | 71% | 68% | 43% | 71% |
| Simon 2024 | 90% | 81% | 75% | 86% | 61% | 100% | 86% |
| Smith 2015 | 100% | 90% | 86% | 90% | 89% | 71% | 86% |
